# Nanoaggregates of Biphilic Carboxyl-Containing Copolymers as Carriers for Ionically Bound Doxorubicin

**DOI:** 10.3390/ma15207136

**Published:** 2022-10-13

**Authors:** Alexander A. Artyukhov, Anna M. Nechaeva, Mikhail I. Shtilman, Evgeniy M. Chistyakov, Alina Yu. Svistunova, Dmitry V. Bagrov, Andrey N. Kuskov, Anca O. Docea, Aristides M. Tsatsakis, Leonid Gurevich, Yaroslav O. Mezhuev

**Affiliations:** 1 Department of Biomaterials, Mendeleev University of Chemical Technology of Russia, 125047 Moscow, Russia; 2 Faculty of Biology, Lomonosov Moscow State University, 119234 Moscow, Russia; 3 Department of Toxicology, Faculty of Pharmacy, University of Medicine & Pharmacy, 2 Petru Rares, 200349 Craiova, Romania; 4 Center of Toxicology Science & Research, Division of Morphology, Medical School, University of Crete, Voutes Campus, 71003 Heraklion, Greece; 5 Department of Materials and Production, Aalborg University, Skjernvej 4A, 9220 Aalborg, Denmark

**Keywords:** targeting, doxorubicin delivery, release kinetics, poly(N-vinyl-2-pyrrolidone-co-acrylic acid) nanoparticles

## Abstract

Application of nanocarriers for drug delivery brings numerous advantages, allowing both minimization of side effects common in systemic drug delivery and improvement in targeting, which has made it the focal point of nanoscience for a number of years. While most of the studies are focused on encapsulation of hydrophobic drugs, delivery of hydrophilic compounds is typically performed via covalent attachment, which often requires chemical modification of the drug and limits the release kinetics. In this paper, we report synthesis of biphilic copolymers of various compositions capable of self-assembly in water with the formation of nanoparticles and suitable for ionic binding of the common anticancer drug doxorubicin. The copolymers are synthesized by radical copolymerization of N-vinyl-2-pyrrolidone and acrylic acid using n-octadecyl-mercaptan as a chain transfer agent. With an increase of the carboxyl group’s share in the chain, the role of the electrostatic stabilization factor of the nanoparticles increased as well as the ability of doxorubicin as an ion binder. A mathematical description of the kinetics of doxorubicin binding and release is given and thermodynamic functions for the equilibrium ionic binding of doxorubicin are calculated.

## 1. Introduction

Pharmacologically active water-soluble drugs are highly bioavailable [1]. At the same time, the rapid distribution of water-soluble drugs in the body reduces the effective concentration of the active drug directly on the pharmacological target and contributes to toxicity [2]. While targeted delivery of drugs poorly soluble in water has been widely addressed with the introduction of nanosized carriers [3,4,5,6] and liposomes [7,8,9], the control of the release of pharmacologically active substances with high water solubility presents significant difficulties [10]. Doxorubicin, which has a wide spectrum of antitumor activity [11,12], is one of the water-soluble anticancer drugs. At the same time, doxorubicin is characterized by high cumulative cardiac toxicity [13,14,15], which limits the possibilities for its use in chemotherapy for tumors.

Rapidly advancing nanomedicine methods make it possible to reduce the cardiac toxicity of doxorubicin by incorporating it into nanoscale aggregates and providing delivery targeting. Nanoparticles of gold [16], iron oxide [17], magnesium aluminum layered double hydroxides [18,19], lipids [20] and oil-in-water microemulsions [21,22] are promising as carriers for doxorubicin loading. Conjugates with biological molecules, such as human serum albumin (HSA), bovine serum albumin (BSA), milk beta-lactoglobulin (β-LG) [23], apoferritin [24] and glycosaminoglycans, as well as platelets [25], have also been studied as potential doxorubicin carriers. Inclusion of doxorubicin into hydrogels can also be used to ensure controlled release [26,27,28]. Another group of carriers is represented by polymers, with which doxorubicin can be bound by either chemically stable [29] or labile covalent bonds in biological systems [30], as well as by hydrogen and ionic bonds [31,32,33,34]. For example, covalent immobilization of doxorubicin through a hydrazine linker on low molecular weight heparin, which has antimetastatic activity, opens up the possibility of a pH-dependent release of the anticancer drug [35].

Although the search for new approaches to the immobilization of doxorubicin continues, the amphiphilic block copolymers consisting of hydrophobic blocks built by residues of lactic and glycolic acids and hydrophilic domains based on polyethylene glycol [36,37], as well as pegylated liposomes [38], including commercial brands Doxil^®^ and Myocet^®^ are considered the gold standards of doxorubicin carriers. However, the use of pegylated block copolymers and liposomes is limited by accelerated clearance associated with the production of PEG-directed immunoglobulins in some patients [39]. The latter circumstance provided the need to search for alternative carriers for doxorubicin free of polyethylene glycol domains. For example, the copolymer of N-vinyl-2-pyrrolidone and allyl glycidyl ether proved to be an effective carrier for the covalent immobilization of doxorubicin to form a copolymer with its own biological activity [29]. Reversible immobilization can be achieved using copolymers of N-(2-hydroxypropyl)methacryl-amide, which form hydrogen bonds with doxorubicin, also due to electrostatic interactions when polyurethanes with carboxyl groups in the side chain are used as polymer carriers [40]. Electrostatic interactions determine the efficiency of graphene oxide nanoparticles in the immobilization of doxorubicin, the surface of which contains predominantly carboxy groups [41]. In general, the electrostatic immobilization of doxorubicin cations on negatively charged nanoparticles is one of the rapidly developing approaches, due to its versatility [40,41,42,43,44,45].

Although many publications indicate the high efficiency of ion binding of doxorubicin [42,43,44,45,46,47,48,49,50], the quantitative side of electrostatic immobilization has not been studied in detail to date. The present article deals with the release kinetics of doxorubicin bound by the coronae of aggregates of the amphiphilic copolymer of N-vinyl-2-pyrrolidone and acrylic acid containing hydrophobic n-octadecyl-thio groups. The introduction of hydrophobic end n-octadecyl-thio groups is achieved by using n-octadecyl-mercaptan as a chain transfer agent. In addition, the use of N-vinyl-2-pyrrolidone residues to form a hydrophilic domain is an alternative to pegylation that is of interest in the treatment of patients capable of producing PEG-directed immunoglobulins.

## 2. Materials and Methods

### 2.1. Materials

Acrylic acid, azobisisobutyro-nitrile and n-octadecyl-mercaptan from Sigma-Aldrich were used. N-vinyl-2-pyrrolidone and 1,4-dioxane were purchased from Himmed. Acrylic acid and N-vinyl-2-pyrrolidone were purified by vacuum distillation; azobisisobutyro-nitrile and n-octadecyl-mercaptan were used without further purification. Doxorubicin hydrochloride was manufactured by Synbias Pharma (Kyiv, Ukraine). Hydro-phthalate buffer (pH = 4), phosphate buffer (pH = 7) and tetraborate buffer (pH = 9) were produced by the “Ekroskhim” company (St. Petersburg, Russia).

### 2.2. Synthesis of Copolymers of N-vinyl-2-pyrrolidone and Acrylic Acid of Various Compositions

10 g (0.09 mol) of N-vinyl-2-pyrrolidone (VP), as well as specified amounts of acrylic acid (AA), n-octadecyl-mercaptan (n-ODM), and azobisisobutyro-nitrile (AIBN) were dissolved in 40 mL of 1,4-dioxane (Table 1).

The copolymerization was carried out for 3 h at 343 K followed by the dilution of the reaction system with 250 mL of distilled water and the evaporation of 1,4-dioxane on a rotary evaporator. Purification of the amphiphilic copolymer was performed by dialysis against distilled water using a 500 MWCO membrane (Labware supplier store) followed by lyophilization (Alpha 1–4 LD plus, Martin Christ, Osterode am Harz, Germany). The precipitated copolymer was further purified from the unreacted n-octadecyl-mercaptan by washing with three 30 mL portions of diethyl ether each.

### 2.3. The Electrostatic (Ionic) Immobilization of Doxorubicin

0.1 g of an amphiphilic copolymer of N-vinyl-2-pyrrolidone and acrylic acid of a given composition was dissolved in 5 mL of a buffer solution with a specified pH. Separately, 0.01 g of doxorubicin hydrochloride was dissolved in 5 mL of a buffer solution with the same pH value. Electrostatic immobilization of protonated doxorubicin was achieved by mixing the prepared solutions.

### 2.4. Doxorubicin Release Kinetics Study

The solutions prepared according to method 2.3 were mixed and placed in a dialysis bag (Labware supplier store 500 MWCO). The kinetics of dialysis at 310 K was studied by measuring absorbance at 480 nm wavelength. The dialysis kinetics were studied in the presence of N-vinyl-2-pyrrolidone copolymers containing 3.9 mol%, 5.6 mol%, 9.8 mol% and 15.8 mol% of acrylic acid residues in the chain (pH = 7).

Separate kinetic measurements were carried out at various concentrations (2.5 × 10^−3^ g^.^mL^−1^; 5.0 × 10^−3^ g^.^mL^−1^; 1.5 × 10^−2^ g^.^mL^−1^) of the N-vinyl-2-pyrrolidone amphiphilic copolymer containing 3.9 mol% of acrylic acid residues in the chain at 310 K (pH = 7).

In addition, the release rates of doxorubicin were determined at 278 K, 298 K and 323 K in the presence of 10^−2^ g^.^mL^−1^ of an amphiphilic N-vinyl-2-pyrrolidone copolymer containing 3.9 mol% acrylic acid residues (pH = 7). The effect of the medium pH on the release rate of doxorubicin was determined in buffer solutions with pH = 4 and pH = 9 at 310 K in the presence of 10^−2^ g^.^mL^−1^ of an amphiphilic N-vinyl-2-pyrrolidone copolymer containing 3.9 mol% acrylic acid residues.

### 2.5. Study Methods

Doxorubicin release kinetics were monitored using a UV-vis spectrophotometer (Eppendorf BioSpectrometer, Hamburg, Germany) and particle size distribution and ζ-potential were determined by dynamic laser light scattering on a NANO-flex II device (Colloid Metrix, Meerbusch, Germany, (Authors express their gratitude to the D.I. Mendeleev Center for the collective use of scientific equipment for assistance in carrying out this research)). The surface tension at the water/air interface was measured by the pendant drop method using the KRUSS DSA30 automated drop shape analyzer at 296 K. Atomic force microscopy (AFM) and transmission electron microscopy (TEM) were used to obtain micrographs of nanoparticles of an amphiphilic N-vinyl-2-pyrrolidone copolymer containing 3.9 mol% acrylic acid residues in the chain.

To prepare the sample for the AFM imaging, freshly cleaved mica sheets were treated with 2.5 mM NiCl_2_ solution in water for 1 min, rinsed and dried. The samples were diluted with water and applied onto the prepared mica sheets. The incubation time was 20–40 s, then the liquid was removed, and the substrate was dried with a stream of air. The measurements were carried out using a Solver PRO-M atomic-force microscope equipped with a Smena scanning head (NT-MDT, Zelenograd, Russian Federation). The NSG10 cantilevers (NT-MDT, Zelenograd, Russian Federation) with a typical force constant k = 11.8 N/m and a curvature radius less than 10 nm were used. Imaging was done in semi-contact mode at 512 × 512 points and 1.2–1.4 Hz scanning frequency. The images were processed using FemtoScan Online Software (Advanced Technologies Center, Moscow, Russian Federation) [51].

For TEM imaging, carbon-coated grids (Ted Pella, Redding, CA, USA) were treated using a glow discharge device Emitech K100X (Quorum Technologies Ltd., Lewes, UK) at 25 mA. The suspension of the nanoparticles (Section 2.3) was deposited onto the grid for 0.5–1 min, then the grids were treated with 1% uranyl acetate for 1–2 min, blotted and dried. Images were obtained using a JEM-1011 (Jeol, Tokyo, Japan) transmission electron microscope equipped with an Orius SC1000W camera (Gatan Inc., Pleasanton, CA, USA). The acceleration voltage was 80 kV; the images were processed using ImageJ software [52]. The TEM measurements were carried at the User Facilities Center “Electron microscopy in life sciences”, Lomonosov Moscow State University (Moscow, Russian Federation).

Cytotoxicity was determined using the MTT assay in vitro on the HepG2 cell line purchased from SPUTNIK (Moscow, Russian Federation) in accordance with the classical method described previously in [53,54].

## 3. Results and Discussion

### 3.1. Nanoparticles of Amphiphilic Copolymers of N-vinyl-2-pyrrolidone and Acrylic Acid of Various Chain Compositions

**Scheme 1:**
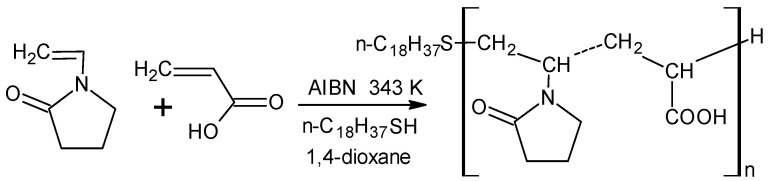
Synthesis of amphiphilic copolymers of N-vinyl-2-pyrrolidone and acrylic acid.

It was previously shown by the ^1^H NMR, ^13^C NMR, IR and MALDI-TOF spectroscopy methods that the radical copolymerization of N-vinyl-2-pyrrolidone and acrylic acid initiated by AIBN in the presence of n-octadecyl-mercaptan yields amphiphilic copolymers (Scheme 1) capable of forming nanosized aggregates in aqueous solutions [50,55].

**Figure 1:**
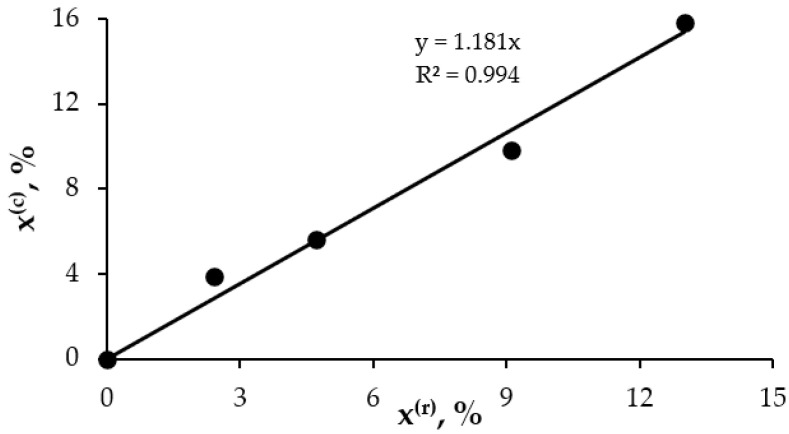
Molar fraction of acrylic acid residues in the amphiphilic copolymer with N-vinyl-2-pyrrolidone (x^(c)^) as a function of the mole fraction of acrylic acid in the reaction mixture (x^(r)^). The yield of N-vinyl-2-pyrrolidone and acrylic acid copolymers after purification by dialysis in all the cases was 65 ± 5%.

In the present publication the amphiphilic copolymers of N-vinyl-2-pyrrolidone containing various amounts of acrylic acid residues were synthesized by varying the N-vinyl-2-pyrrolidone/(acrylic acid) ratio in the initial reaction mixture (Scheme 1). As can be seen in Figure 1, the copolymer is enriched with acrylic acid residues when its mole fraction in the reaction system is up to 13 mol% after copolymerization to a deep conversion of comonomers and subsequent purification by dialysis. In this case, dialysis purification can lead to a composition of the resulting amphiphilic copolymers different from that prescribed by the copolymerization constants.

The number-average molecular weight of the random copolymers was determined by end group analysis [55], which was about 6500 ± 500 regardless of the N-vinyl-2-pyrrolidone/(acrylic acid) ratio in the initial reaction mixture.

**Figure 2:**
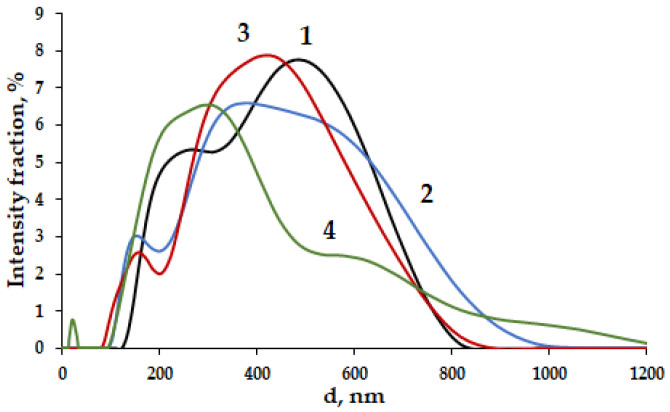
Intensity diameter distributions of particles formed as a result of self-assembly of the chains of amphiphilic copolymers of N-vinyl-2-pyrrolidone and acrylic acid with a mole fraction of acrylic acid residues as follows: 1—3.9 mol%; 2—5.6 mol%; 3—9.8 mol%; 4—15.8 mol%.

At the same time, the intensity diameter distribution of nanoparticles is very sensitive to the mole fraction of acrylic acid residues in the chain (Figure 2).

**Figure 3:**
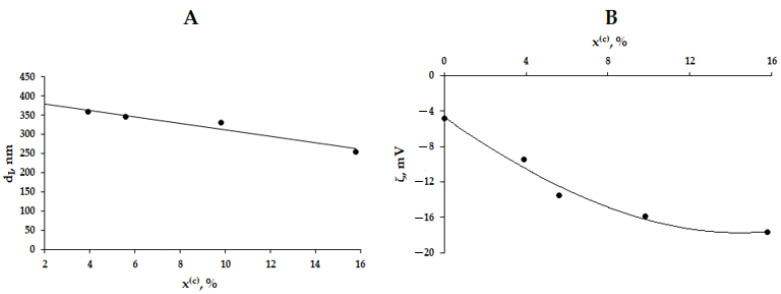
(**A**) Intensity-average diameter ${\bar{d}}_{I}$ and (**B**) electrokinetic potential ζ of the amphiphilic of N-vinyl-2-pyrrolidone and acrylic acid copolymer aggregates as a function of the mole fraction of acrylic acid residues in the chain (x^(c)^).

The intensity-average particle diameter tends to decrease with the increase in the mole fraction of acrylic acid residues in the polymer chain (Figure 2 and Figure 3A), which is a consequence of a natural decrease in the electrokinetic potential (Figure 3B). An increase in the contribution of the electrostatic stabilization of aggregates caused the appearance of a separate fraction of particles ranging from 12 nm to 40 nm in diameter for the N-vinyl-2-pyrrolidone copolymer containing 15.8 mol% acrylic acid residues (Figure 2, curve 4). The PDI was 0.176, 0.281, 0.215 and 0.359 for nanoparticles formed by chains of amphiphilic copolymers of N-vinyl-2-pyrrolidone containing 3.9, 5.6, 9.8 and 15.8 mol% acrylic acid residues, respectively.

**Figure 4:**
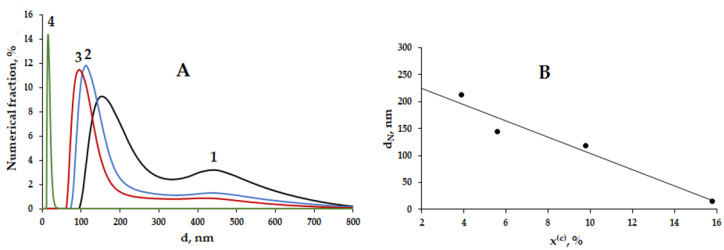
(**A**) Numerical diameter distributions of particles formed as a result of self-assembly of chains of the amphiphilic copolymers of N-vinyl-2-pyrrolidone and acrylic acid with a mole fraction of acrylic acid residues as follows: 1—3.9 mol%; 2—5.6 mol%; 3—9.8 mol%; 4—15.8 mol%; (**B**) Numerical -average diameter ${\bar{d}}_{N}$ as a function of the mole fraction of acrylic acid residues in the chain.

The electrostatic stabilization effect of the nanoparticles with an increase in the mole fraction of acrylic acid residues in the chain of the amphiphilic copolymer was consistent with the curves of the numerical distribution of particle diameters (Figure 4A). As can be seen, the number average particle diameter was much more sensitive to changes in the share of carboxyl groups in the chain (Figure 4B) than the intensity-average diameter (Figure 3A). In addition, for the copolymer containing 15.8 mol% of acrylic acid residues there was no pronounced peak above 40 nm on the curves of the numerical distribution of particle diameters. Thus, the system contains a significant number of nanoparticles under 40 nm in diameter along with large aggregates, the number of which is small, while the amphiphilic copolymer mass contained therein is significant. On the contrary, for particles containing 3.9 mol% acrylic acid residues, the diameter distribution had a pronounced bimodal character, which is associated with insufficient electrostatic stabilization of the nanoparticles (Figure 4A).

**Figure 5:**
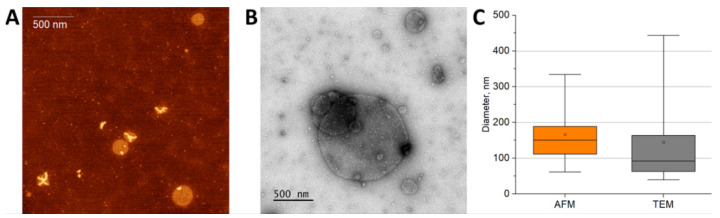
Characterization of the particles using AFM (**A**) and TEM (**B**). In the AFM image the vertical range is 10 nm. (**C**) Box-plot showing the particle diameter distribution. The plot shows the median (line), the 25th–75th percentile range (box), the 5th–95th percentile range (whiskers) and the mean (dot).

The nanoparticles were imaged using AFM and TEM (Figure 5A,B, correspondingly). The round-shaped particles could be seen clearly, although some aggregates were also visible. Figure 5C summarizes the information on the individual diameters. The data obtained with AFM and TEM were in reasonable agreement, and the mean values (167 ± 26 nm and 145 ± 24 nm, respectively, mean ± CI) were close to the position of the first peak of the number distribution obtained using DLS (Figure 5A). In the AFM images, the height of the particles was in the range from 2.5 to 8 nm, far smaller than the mean diameter (167 nm). The mismatch between the height and the lateral size indicates flattening or spreading of the copolymer over the substrate. Due to its small amount, the ionic immobilization of doxorubicin did not significantly affect the particle diameter, as noted earlier [50]. As can be seen, the dynamic laser light scattering method showed the existence of a fraction of particles with a larger diameter than what follows from the results of AFM and TEM. The latter indicates the existence of a dense hydration shell associated with the hydrophilic corona of nanoparticles.

**Figure 6:**
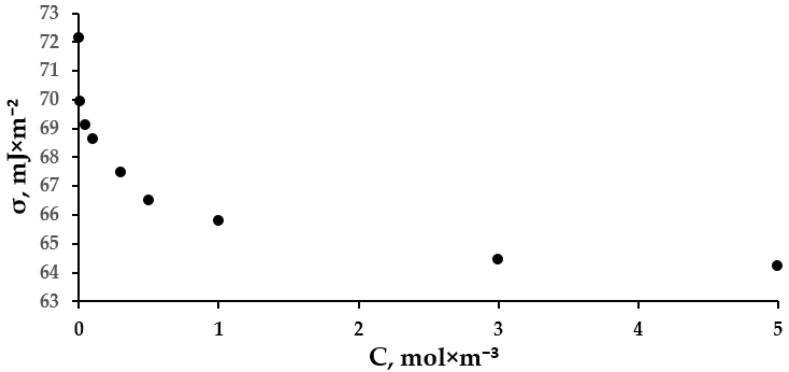
The surface tension isotherm of the amphiphilic copolymer of N-vinyl-2-pyrrolidone and acrylic acid (x^(c)^ = 3.9 mol%).

The obtained amphiphilic copolymers exhibited surface activity. For example, a copolymer containing 3.9 mol% acrylic acid lowered the surface tension at the water/air interface down to 64 mJ^.^m^−2^ already at 5 mol^.^m^−3^ (Figure 6).

### 3.2. Theoretical Consideration of the Release Kinetics of Doxorubicin Immobilized by an Amphiphilic Copolymer of N-vinyl-2-pyrrolidone and Acrylic Acid

**Scheme 2:**
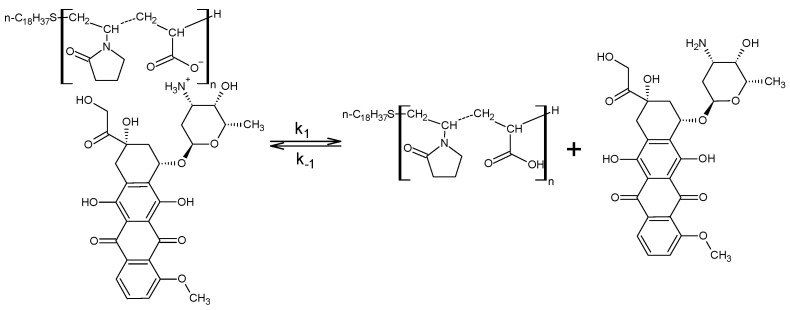
Release mechanism of immobilized doxorubicin.

**Figure 7:**
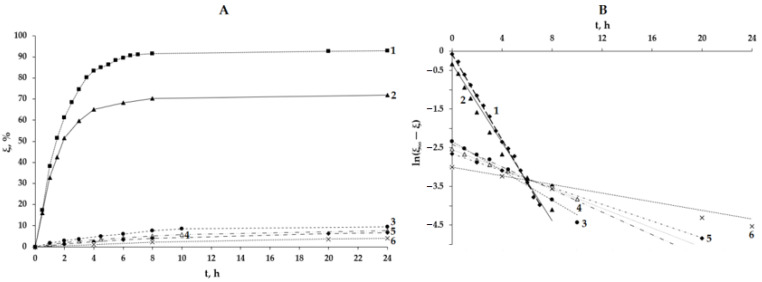
(**A**) Kinetic curves of doxorubicin release and (**B**) their linear anamorphoses in the “$\ln\left(\xi_{\infty }-\xi \right)$ vs. t” coordinates in the presence of N-vinyl-2-pyrrolidone copolymers containing various mole fractions of acrylic acid residues: 1—in the absence of copolymer additives; 2—0% (N-vinyl-2-pyrrolidone homopolymer); 3—3.9 mol%; 4—5.6 mol%; 5—9.8 mol%; 6—15.8 mol% (ξ and $\xi_{\infty }$ are the conversion and equilibrium conversion of doxorubicin release; t —time).

Apparently, the immobilization of doxorubicin is associated with electrostatic interionic interactions involving the carboxy group of acrylic acid residues, since the amphiphilic N-vinyl-2-pyrrolidone homopolymer only slightly slowed down the release of doxorubicin (Figure 7A). Thus, one may assume that an equilibrium is established as shown in Scheme 2.

The kinetics of the equilibration presented in Scheme 2 can be described by Equation (1).

(1) $$\frac{dC_{DOX}}{dt}=k_{1}C_{IDOX}-k_{-1}^{\prime }C_{COOH}C_{DOX}=k_{1}C_{IDOX}-k_{-1}C_{DOX}$$

where: $C_{IDOX}$—concentration of doxorubicin immobilized through electrostatic interactions; $C_{COOH}$—concentration of acrylic acid residues in the system; $C_{DOX}=C_{DOX}^{max}-C_{IDOX}$—concentration of free doxorubicin; $C_{DOX}^{max}$—the maximum concentration of doxorubicin that would have been achieved upon its complete release; $k_{1}$, $k_{-1}=k_{-1}^{\prime }C_{COOH}$—doxorubicin release and binding rate constants; $k_{-1}^{\prime }$—doxorubicin binding rate constant normalized to the concentration of carboxyl groups; t—time (see Supplementary Information).

After separating the variables and integrating the left- and right-hand sides of the differential equation from 0 to $C_{DOX}$ and from 0 to *t*, respectively, Equation (2) can be derived.

(2) $$\ln\left(1-\frac{C_{DOX}}{\bar{C_{DOX}}}\right)=-\left(k_{1}+k_{-1}\right)t$$

where: $\bar{C_{DOX}}$ is the equilibrium concentration of free doxorubicin. Assuming that the conversion and the equilibrium release conversion of doxorubicin can be represented by Equations (3) and (4), respectively, after substitution into Equation (2), Equation (5) can be obtained.

(3) $$\xi =\frac{C_{DOX}}{C_{DOX}^{max}}$$

(4) $$\xi_{\infty }=\frac{\bar{C_{DOX}}}{C_{DOX}^{max}}$$

(5) $$\ln\left(\xi_{\infty }-\xi \right)=\ln\xi_{\infty }-\left(k_{1}+k_{-1}\right)t$$

where: ξ and $\xi_{\infty }$ are the conversion and equilibrium conversion of doxorubicin release.

When equilibrium is reached, the concentration of free doxorubicin remains constant over time; therefore, the differential Equation (1) can be transformed into the algebraic Equation (6):

(6) $$K=\frac{k_{1}}{k_{-1}}=\frac{\bar{C_{DOX}}}{\bar{C_{IDOX}}}=\frac{\bar{C_{DOX}}\;}{C_{DOX}^{max}-\bar{C_{DOX}}\;}=\frac{\xi_{\infty }\;}{1-\xi_{\infty }\;}$$

Equation (6) allows calculation of the doxorubicin release equilibrium constant as a result of the experimental determination of $\xi_{\infty }$. On the other hand, linear dependencies in the coordinates “$\ln\left(\xi_{\infty }-\xi \right)$ vs. *t*” allow determination of the sum of the rate constants $k=k_{1}+k_{-1}$ in accordance with Equation (5). Thus, the rate constants $k_{1}$ and $k_{-1}$ can be found separately according to the Equations (7) and (8).

(7) $$k_{1}=\frac{kK}{1+K}$$

(8) $$k_{-1}=\frac{k}{1+K}$$

The binding rate constant of doxorubicin taken relative to the concentration of carboxy groups ($k_{-1}^{\prime }$) can be calculated in accordance with Equation (9):

(9) $$k_{-1}^{\prime }=\frac{k_{-1}}{C_{COOH}}=\frac{K}{\left(1+K\right)C_{COOH}}$$

The resulting ratios (1), (2), and (5) are similar to the kinetic equations describing first-order reversible chemical reactions [56].

### 3.3. Kinetics of the Release of Doxorubicin Corona-Bound on the Nanoparticles Formed by Chains of Amphiphilic Copolymer of N-vinyl-2-pyrrolidone and Acrylic Acid

The rate and the equilibrium conversion of doxorubicin release decreased with the increase in the molar fraction of acrylic acid residues in the amphiphilic copolymer chain (Figure 7A). The resulting kinetic curves were linear in the “$\ln\left(\xi_{\infty }-\xi \right)$ vs. *t*” coordinates (Figure 7B), which indicates the relevance of Equations (5) and (6) to the experimental data. Doxorubicin release rate constants ($k_{1}$) were 1.65 × 10^−2^ h^−1^, 1.03 × 10^−2^ h^−1^, 7.58 × 10^−3^ h^−1^ and 2.80 × 10^−3^ h^−1^ in the presence of the amphiphilic copolymers of N-vinyl-2-pyrrolidone (10^−2^ g^.^mL^−1^) containing 3.9 mol%, 5.6 mol%, 9.8 mol% and 15.8 mol% of acrylic acid residues in the chain. The doxorubicin binding rate constants ($k_{-1}$) were 0.153 h^−1^, 0.118 h^−1^, 0.101 h^−1^ and 0.053 h^−1^ in the presence of the amphiphilic copolymers of N-vinyl-2-pyrrolidone (10^−2^ g^.^mL^−1^) containing 3.9 mol%, 5.6 mol%, 9.8 mol% and 15.8 mol% of acrylic acid residues in the chain, respectively.

**Figure 8:**
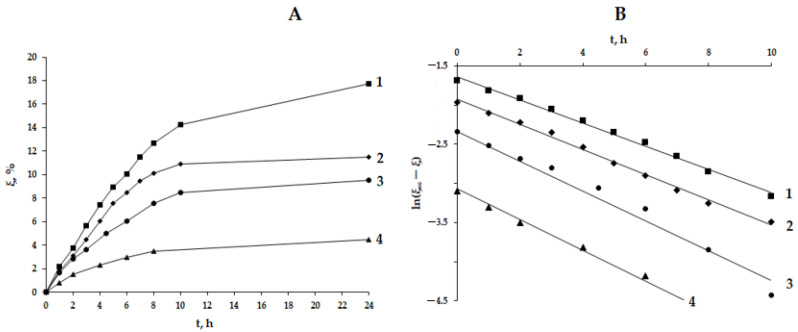
(**A**) Kinetic curves of release of doxorubicin and (**B**) their linear anamorphoses in the “$\ln\left(\xi_{\infty }-\xi \right)$ vs. *t*” coordinates in the presence of N-vinyl-2-pyrrolidone and acrylic acid copolymers (x^(c)^ = 3.9 mol%) of various concentrations: 1—2.5 × 10^−3^ g^.^mL^−1^; 2—5.0 × 10^−3^ g^.^mL^−1^; 3—10^−2^ g^.^mL^−1^; 4—1.5 × 10^−2^ g^.^mL^−1^ (ξ and $\xi_{\infty }$ are the conversion and equilibrium conversion of doxorubicin release; t —time).

An increase in the concentration of the amphiphilic N-vinyl-2-pyrrolidone copolymer containing 3.9 mol% of acrylic acid also slowed the release of doxorubicin and reduced the equilibrium conversion of this process (Figure 8A). The experimental kinetic curves also remain linear in the “$\ln\left(\xi_{\infty }-\xi \right)$ vs. *t*” coordinates (Figure 8B), which makes it possible to calculate all kinetic parameters in accordance with the Equations (5)–(8). Doxorubicin release rate constants ($k_{1}$) were 2.56 × 10^−2^ h^−1^, 2.16 × 10^−2^ h^−1^, 1.65 × 10^−2^ h^−1^ and 8.77 × 10^−3^ h^−1^ in the presence of amphiphilic N-vinyl-2-pyrrolidone and acrylic acid copolymers (x^(c)^ = 3.9 mol%) of various concentrations: 2.5 × 10^−3^ g^.^mL^−1^; 5.0 × 10^−3^ g^.^mL^−1^; 10^−2^ g^.^mL^−1^; 1.5 × 10^−2^ g^.^mL^−1^. Doxorubicin binding rate constants ($k_{-1}$) were 0.113 h^−1^, 0.133 h^−1^, 0.153 h^−1^ and 0.186 h^−1^ in the presence of N-vinyl-2-pyrrolidone and acrylic acid copolymers (x^(c)^ = 3.9 mol%) of various concentrations: 2.5 × 10^−3^ g^.^mL^−1^; 5.0 × 10^−3^ g^.^mL^−1^; 10^−2^ g^.^mL^−1^; 1.5 × 10^−2^ g^.^mL^−1^.

**Figure 9:**
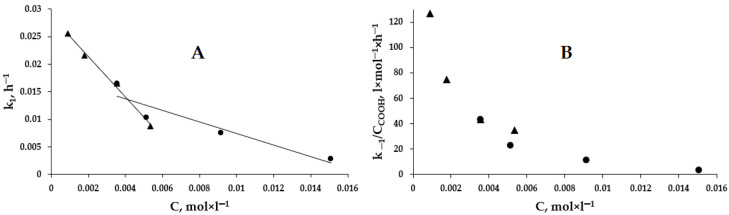
The rate constants of doxorubicin (**A**) release ($k_{1}$) and (**B**) binding ($k_{-1}^{\prime }=\frac{k_{-1}}{C_{COOH}}$ ) as a function of the concentration of acrylic acid residues in the system (triangles—change in $C_{COOH}$ due to an increase in the concentration of the N-vinyl-2-pyrrolidone amphiphilic copolymer containing 3.9 mol% acrylic acid residues; circles—change in $C_{COOH}$ due to an increase in the mole fraction of acrylic acid residues in the amphiphilic copolymer at 10^−2^ g^.^mL^−1^).

Thus, with an increase in the concentration of carboxy groups in the system both due to an increase in the mole fraction of acrylic acid residues in the chain of the amphiphilic copolymer and as a result of an increase in the copolymer concentration a decrease in the rate constant of doxorubicin release ($k_{1}$) was observed. In both cases, the decrease in $k_{1}$ with an increase in the concentration of acrylic acid residues is apparently associated with an increase in the density of the negative charge of nanoparticles and an increase in electrostatic interaction with doxorubicin cations, at which $k_{1}$ decreased faster due to the growth of the carboxy groups concentration along with the copolymer concentration increase than upon varying its composition (Figure 9A). The latter effect is apparently associated with the electrostatic repulsion of doxorubicin cations when negatively charged carboxy groups (immobilization centers) are located within the same chain. The doxorubicin binding rate constant taken relative to the concentration of carboxy groups ($k_{-1}^{\prime }$) also decreased with an increase in the concentration of acrylic acid residues, which can be explained by a decrease in the degree of dissociation of carboxy groups due to association of ions (Figure 9B).

**Figure 10:**
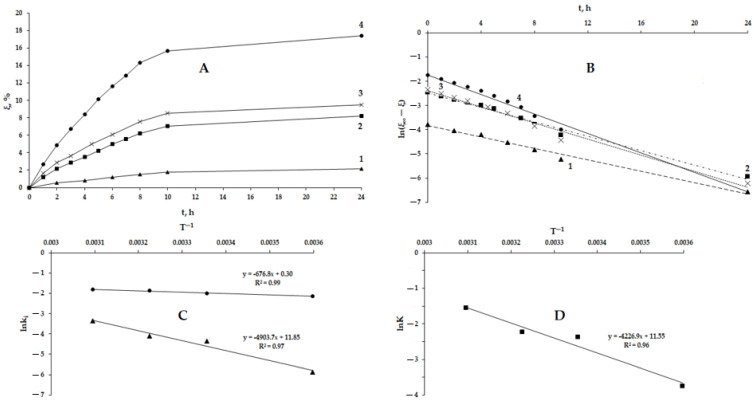
(**A**) Kinetic curves for doxorubicin release and (**B**) their linear anamorphoses in the “$\ln\left(\xi_{\infty }-\xi \right)\;$vs. t” coordinates in the presence of a copolymer of N-vinyl-2-pyrrolidone and acrylic acid (x^(c)^ = 3.9 mol%; copolymer concentration 10^−2^ g^.^mL^−1^) at the following temperatures: 1—278 K, 2—298 K, 3—310 K and 4—323 K. The temperature dependences of (**C**) the rate constants $k_{1}$ (triangles) and $k_{-1}$ (circles) and (**D**) the equilibrium constants (K) (ξ and $\xi_{\infty }$ are the conversion and equilibrium conversion of doxorubicin release; t —time).

The rate and the equilibrium conversion of doxorubicin release increased with the temperature increase (Figure 10A) and the kinetic data were linear in the “$\ln\left(\xi_{\infty }-\xi \right)$ vs. t” coordinates (Figure 10B). The $k_{1}$ values were 2.80 × 10^−3^ h^−1^; 1.27 × 10^−2^ h^−1^; 1.65 × 10^−2^ h^−1^; 3.52 × 10^−2^ h^−1^ at 278 K, 298 K, 310 K and 323 K, respectively. The $k_{-1}$ were 0.119 h^−1^; 0.137 h^−1^; 0.153 h^−1^; 0.166 h^−1^ at 278 K, 298 K, 310 K and 323 K, respectively. Therefore, the activation energy of the doxorubicin release process was 40.7 kJ^.^mol^−1^, while the activation energy of doxorubicin binding was 5.6 kJ^.^mol^−1^ (Figure 10C). Thus, the doxorubicin release rate increased rapidly with rising temperature and the reaction responsible for the release of doxorubicin proceeds in the kinetic region. On the contrary, the binding rate of doxorubicin is almost independent of temperature and occurs in the diffusion region. Since $k_{1}$ increased faster than $k_{-1}$ with temperature growth, the equilibrium constant for the doxorubicin release (K) increased with growing temperature (Figure 10D).

The standard enthalpy of doxorubicin release determined from the slope of the straight line in Figure 10D was practically independent of temperature in the temperature range of 278 K–323 K and was about 35 kJ^.^mol^−1^. The changes in the standard Gibbs energy and standard entropy for the doxorubicin release process at 298 K were 5.9 kJ^.^mol^−1^ and 98 J^.^mol^−1.^K^−1^, respectively, and were calculated using Equations (10) and (11).

(10) $$\Delta G_{T}^{0}=-RTlnK$$

(11) $$\Delta S_{T}^{0}=\frac{\Delta H_{T}^{0}-\Delta G_{T}^{0}}{T}$$

**Figure 11:**
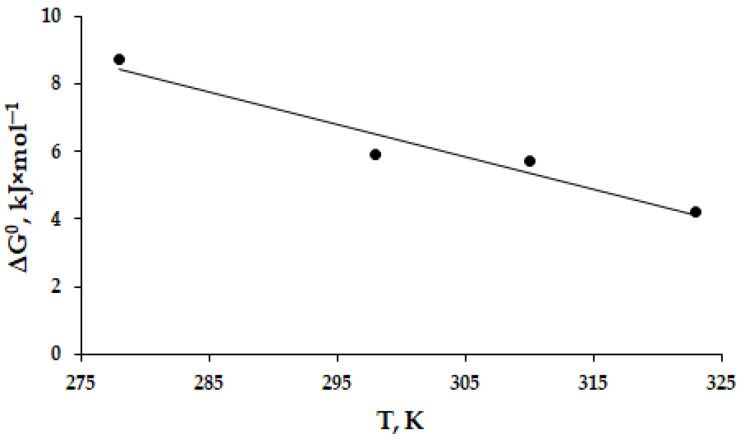
Standard Gibbs free energy change for doxorubicin release as a function of temperature.

Although the changes in the standard enthalpy and standard entropy during the release of doxorubicin are almost independent of temperature in the range of 278 K–323 K, the change in the standard Gibbs energy of doxorubicin release decreased linearly with the growth of temperature (Figure 11).

A positive change in the standard entropy during the release of doxorubicin indicates the formation of a neutral uncharged carboxy group and the main form of doxorubicin (Scheme 2), since the formation of ions would lead to structuring of water molecules and would be accompanied by only a small increase in entropy or even the decrease thereof. The reasons for the positive change in the standard entropy upon the release of doxorubicin are an increase in the number of particles in the solution, as well as the presumed disappearance of the particle charge (Scheme 2), which has an ordering effect on the surrounding water molecules. The positive value of the standard enthalpy change for the doxorubicin release process is a consequence of the endothermic dissociation of the protonated forms of primary amines [57].

**Figure 12:**
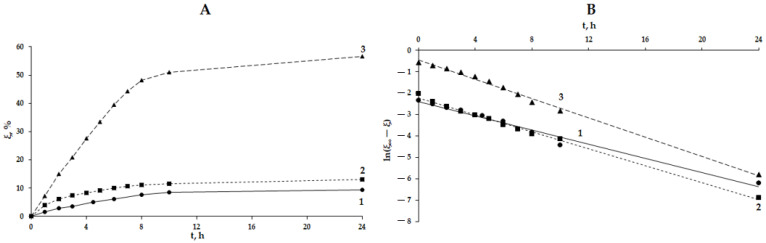
(**A**) Kinetic curves of doxorubicin release and (**B**) their linear anamorphoses in the “$\ln\left(\xi_{\infty }-\xi \right)\;$vs. *t*” coordinates in the presence of a copolymer of N-vinyl-2-pyrrolidone and acrylic acid (x(c) = 3.9 mol%; copolymer concentration 10^−2^ g^.^mL^−1^) in buffer solutions: 1—pH = 7; 2—pH = 9; 3—pH = 4. Temperature = 310 K (ξ and $\xi_{\infty }$ are the conversion and equilibrium conversion of doxorubicin release; t —time).

Thus, the electrostatic immobilization of doxorubicin is an equilibrium immobilization. Therefore, the proportion of doxorubicin immobilized in equilibrium with free doxorubicin is determined by the specific release conditions. The share of free doxorubicin can be defined as the asymptote towards which the release kinetic curves tend shown in Figure 7A, Figure 8A, Figure 10A and Figure 12A. The experimentally determined doxorubicin binding efficiency is about 92% under conditions close to physiological (310 K, pH = 7, Figure 10A).

The transition from a neutral medium to either alkaline or acidic led to an increase in the doxorubicin release rate (Figure 12). Thus, in a buffer with pH = 9 the rate constants were $k_{1}=$ 0.0282 h^−1^ and $k_{-1}=$ 0.185 h^−1^, while at pH = 7 these rate constants were $k_{1}=$ 0.0165 h^−1^ and $k_{-1}=$ 0.153 h^−1^ at 310 K. At pH = 4 the rate constants were $k_{1}=$ 0.123 h^−1^ and $k_{-1}=$ 0.093 h^−1^ at 310 K.

**Scheme 3:**
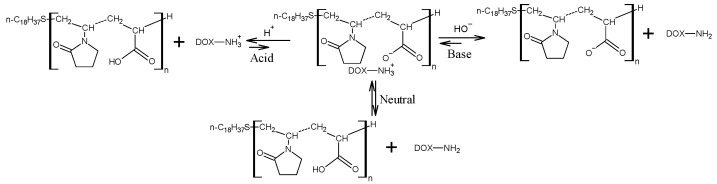
The doxorubicin release mechanisms in various media.

The increase in the drug release rate in an acidic or alkaline medium is a consequence of the electrostatic mechanism of acid-base doxorubicin immobilization. Apparently, in an acidic medium doxorubicin is released in the salt form due to its displacement by protons, and in the basic form in an alkaline medium due to deprotonation of the doxorubicin cation. In close-to-neutral media the drug release occurs due to proton transfer from the doxorubicin cation to the carboxylate anions of acrylic acid residues, as was established above (Scheme 3).

Thus, the pH effect on the drug release rate is associated with an increase in the proportion of non-immobilized doxorubicin in the salt (in an acidic medium) or basic form (in an alkaline medium). On the other hand, it is known that cancer cells are often characterized by a lower pH value compared to normal cells [58,59]. However, the main difference between cancer cells and normal cells is the high intensity of metabolic processes and an increased rate of endocytosis, which is realized through specific mechanisms such as macro-pinocytosis [60]. Since in endosomes the medium pH is 4–5 [61], it can be expected that an increased rate of doxorubicin release in acidic media (Figure 12, curve 3) combined with an increased intensity of endocytosis in cancer cells can serve as a targeting factor. In general, high pH sensitivity is characteristic of the release rate of doxorubicin bound to the carrier through electrostatic interactions [62,63]. The time it takes to reach equilibrium release of electrostatically immobilized doxorubicin by an amphiphilic copolymer of N-vinyl-2-pyrrolidone and acrylic acid is close to that determined in works [21,22,63] when gold nanoparticles stabilized with pectin (galacturonic acid) [63] or microemulsions were used as carriers [21,22]. The application of a pegylated copolymer of lactic and glycolic acids described in [37] makes it possible to increase the time to reach the equilibrium release of doxorubicin, but it is complicated by a pulsed release of about 20% of the drug at the initial time. As can be seen (Figure 7A, Figure 8A, Figure 10A and Figure 12A), the use of electrostatic immobilization of doxorubicin with aggregates of the amphiphilic copolymer of N-vinyl-2-pyrrolidone and acrylic acid makes it possible to prevent the pulsed release of the drug. Although electrostatic interactions are a basis for the immobilization of doxorubicin by many various carriers [40,41,42,43,44,45,46,47,48,49,50], to the best of the authors’ knowledge a detailed quantitative analysis of the kinetic patterns of drug release in similar systems, is considered herein for the first time.

It is known that the cytotoxicity of copolymers of N-vinyl-2-pyrrolidone and ethylene oxide increases with an increase in the molar fraction of acrylic acid residues in the chain [64,65]. The survival rate of HepG2 cells in the presence of nanoparticles formed by chains of the amphiphilic N-vinyl-2-pyrrolidone copolymer containing 3.9 mol% acrylic acid residues at a concentration of 1 mg^.^mL^−1^ in water according to the MTT test was more than 95%. Thus, the obtained results indicate a low cytotoxicity of the amphiphilic copolymer of N-vinyl-2-pyrrolidone and acrylic acid, which is consistent with the literature data [53,66,67,68]. On the other hand, both the aqueous solution of doxorubicin hydrochloride with a concentration of 0.1 mg^.^mL^−1^ and the same concentration of doxorubicin electrostatically immobilized on amphiphilic copolymer of N-vinyl-2-pyrrolidone and acrylic acid with a concentration of 1 mg^.^mL^−1^ showed the survival rate of HepG2 cells less than 20%. This result is consistent with the sensitivity of HepG2 cells to doxorubicin [69,70]. Therefore, the electrostatic binding of doxorubicin does not reduce the effectiveness of its antitumor effect. At the same time, the noted effect of an increase in the release rate of doxorubicin with a decrease in the pH of the medium can contribute to the accumulation of the antitumor drug in cancer cells.

Nanoparticles formed by an amphiphilic copolymer of N-vinyl-2-pyrrolidone containing 3.9 mol% acrylic acid at a carrier concentration of 10^−2^ g^.^mL^−1^ and 1 mg^.^mL^−1^ doxorubicin retain colloidal stability in aqueous media for 72 h and probably for much longer. At the same time, the recommended form of drug storage is a solid dispersion of doxorubicin and carrier copolymer, which redisperses in water to form stable nanoparticles. Thus, the dosage form can be prepared immediately before administration to the body, which also allows varying the therapeutic dose of doxorubicin.

## 4. Conclusions

It is shown that amphiphilic copolymers of N-vinyl-2-pyrrolidone and acrylic acid are capable of reversible immobilization of protonated doxorubicin. The immobilization efficacy is proportional to the molar fraction of acrylic acid residues in the system. The decrease in the doxorubicin release rate constant is found to be more affected by the increase in the concentration of carboxyl groups of acrylic acid residues with an increase in the copolymer concentration as compared to the composition change. We attribute this to electrostatic repulsion of doxorubicin cations when the immobilization centers are located in the same chain. The kinetics of doxorubicin release in all the cases studied obeys the equation for reversible first-order reactions. The standard enthalpy and standard entropy of the doxorubicin release process are +35 kJ·mol^−1^ and +98 J·mol^−1^·K^−1^, respectively. Thus, binding of the protonated form of doxorubicin is of an electrostatic nature, and the release of doxorubicin is associated with deprotonating and the formation of uncharged carboxyl and amino groups (pH close to neutral). The release rate of doxorubicin significantly increases with a decrease in the pH of the medium, which can serve as the most important factor in targeted delivery of doxorubicin, and as a result lead to the reduction of its the side effect, in particular cardiotoxicity. The results of the present study may be of interest for quantitative modeling of the release kinetics of electrostatically immobilized doxorubicin from other nanoparticles, as well as for the creation of new nanosized doxorubicin carriers that are promising for the treatment of patients capable of producing PEG-directed immunoglobulins. However, further in vivo studies are needed to determine the cardiac toxicity and targeting potential of application of amphiphilic copolymers of N-vinyl-2-pyrrolidone and acrylic acid as doxorubicin carriers. In addition, acrylic acid residues can be used as a site for vectorization of the doxorubicin carrier by click chemistry methods.

## Figures and Tables

**Table 1:** Amounts of substances used in the synthesis of amphiphilic copolymers of N-vinyl-2-pyrrolidone (VP) and acrylic acid (AA) of various compositions (x^(r)^ being the mole fraction of acrylic acid in the reaction mixture; n-ODM—n-octadecyl-mercaptan; AIBN—azobisisobutyro-nitrile).

x^(r)^, mol%	AA, g	n-ODM, g	AIBN, g
0	0	0.257	0.100
2.4	0.162	0.258	0.102
4.7	0.323	0.259	0.103
9.1	0.646	0.260	0.106
13	0.969	0.261	0.110

## Data Availability

All data obtained by direct measurement are given in the manuscript and are also available from the corresponding author.

## Supplementary Materials

The following supporting information can be downloaded at: https://www.mdpi.com/article/10.3390/ma15207136/s1, Nomenclature of used abbreviations and values.
