# Supplementary material for: Nanoaggregates of Biphilic Carboxyl-Containing Copolymers as Carriers for Ionically Bound Doxorubicin
Source: Materials (Basel). 2022 Oct 13;15(20):7136. doi: 10.3390/ma15207136 (PMC9609473; doi:10.3390/ma15207136)
Supplement: Supplementary file 1 [file materials-15-07136-s001.zip › materials-1930974-supplementary.pdf]

Supplementary Materials

Nomenclature of used abbreviations and values

AA – acrylic acid;

AFM – atomic force microscopy;

AIBN – azobisisobutyronitrile;

C_{IDOX} – concentration of doxorubicin immobilized through electrostatic interactions;

C_{COOH} – concentration of acrylic acid residues in the system;

$C_{DOX} = C_{DOX}^{max} - C_{IDOX}$ – concentration of free doxorubicin;

C_{DOX}^{max} – the maximum concentration of doxorubicin that would have been achieved upon its complete release;

$\overline{C_{DOX}}$ is the equilibrium concentration of free doxorubicin;

ΔG_T^0 – standard Gibbs energy of doxorubicin release;

ΔH_T^0 - standard enthalpy of release of doxorubicin;

k_1- doxorubicin release rate constant;

$k_{-1} = k'_{-1}C_{COOH}$ – doxorubicin binding rate constant;

k'_{-1} – doxorubicin binding rate constant normalized to the concentration of carboxyl groups;

K - doxorubicin release equilibrium constant;

n-ODM – n-octadecylmercaptan;

ΔS_T^0 – standard entropy of release of doxorubicin;

TEM – transmission electron microscopy;

t – time;

VP – N-vinyl-2-pyrrolidone;

$x^{(r)}$ – mole fraction of acrylic acid in the reaction mixture;

$x^{(c)}$ – mole fraction of acrylic acid in the copolymer;

ξ and ξ_∞ are the conversion and equilibrium conversion of doxorubicin release.
